# Cellulose degradation by oxidative enzymes

**DOI:** 10.5936/csbj.201209015

**Published:** 2012-11-09

**Authors:** Maria Dimarogona, Evangelos Topakas, Paul Christakopoulos

**Affiliations:** a BIOtechMASS Unit, Biotechnology Laboratory, School of Chemical Engineering, National Technical University of Athens, 5 Iroon Polytechniou Str, Zografou Campus, 15700, Athens, Greece; b Biochemical and Chemical Process Engineering, Division of Sustainable Process Engineering, Department of Civil, Environmental and Natural Resources Engineering, Luleå University of Technology,SE-97187Luleå, Sweden

**Keywords:** GH61, polysaccharide monooxygenases, CBM33, biofuels, bioethanol, cellulose

## Abstract

Enzymatic degradation of plant biomass has attracted intensive research interest for the production of economically viable biofuels. Here we present an overview of the recent findings on biocatalysts implicated in the oxidative cleavage of cellulose, including polysaccharide monooxygenases (PMOs or LPMOs which stands for lytic PMOs), cellobiose dehydrogenases (CDHs) and members of carbohydrate-binding module family 33 (CBM33). PMOs, a novel class of enzymes previously termed GH61s, boost the efficiency of common cellulases resulting in increased hydrolysis yields while lowering the protein loading needed. They act on the crystalline part of cellulose by generating oxidized and non-oxidized chain ends. An external electron donor is required for boosting the activity of PMOs. We discuss recent findings concerning their mechanism of action and identify issues and questions to be addressed in the future.

## 1. Introduction

The increasing global demand for energy, coupled with diminishing reserves and global warming have made imperative the gradual replacement of fossil fuels by alternative resources such as renewable energies [1]. Among these, biomass is one of the most promising sources for the production of transportation fuels. Biomass-derived ethanol is currently the most widely used biofuel in the United States and is mainly produced from starch or sugar [2]. However, since the latter are also food sources, the production of second-generation bioethanol, mainly derived from lignocellulosic feedstocks, has been a goal for government and private industry for the last three decades [3]. The conversion of lignocellulosics to ethanol involves two processes: degradation of biomass to fermentable sugars, usually catalyzed by cellulolytic enzymes, and fermentation of the sugars to ethanol by yeasts or bacteria. Depending on the composition of the starting material, various pretreatment techniques have been developed in order to prepare it for the subsequent step of enzyme hydrolysis [4]. One of the main obstacles for the financially competitive production of ethanol has been the high cost of both pretreatment and hydrolysis steps, resulting from the increased biomass recalcitrance [5]. Dedicated efforts have been therefore focused on the development of cost-effective and robust biocatalysts used for breaking down lignocellulose to fermentable sugars.

Lignocellulosic biomass is mainly composed of plant cell walls that vary substantially in their contents depending on the species, variety and climate. Their main component is cellulose, the most abundant natural polymer on earth. The primary structure of cellulose is an unbranched glucan chain of repeating β-(1,4)-D glucose units. Many parallel glucans snap into crystalline microfibrils. Native cellulose occurs in two different crystal forms, a single-chain triclinic phase (I*α*) and a two-chain monoclinic phase (I*β*) [6] and is highly resistant to enzymatic attack [7]. Cellulosic fibrils are embedded in a complex matrix involving hemicelluloses and lignin that hamper the way to cellulases and hemicellulases. Hemicelluloses are heterogeneous polymers of pentoses (e.g. xylose and arabinose), hexoses (e.g. mannose, glucose and galactose) and sugar acids (e.g. acetic, galacturonic and glucuronic). Contrary to cellulose, hemicelluloses are random and amorphous and more easily degraded to single sugars [8]. Hardwood hemicellulases contain mainly xylans, while softwood hemicellulases contain mainly glucomannans. Lignin is a complex aromatic polymer constructed of phenylpropane units derived from sinapyl, *p*-coumaryl and coniferyl alcohol. Lignin, hemicellulose and cellulose are linked by chemical bonds, forming a complex matrix that hampers the way to hemicellulases and cellulases [9, 10].

Plant biomass degradation by fungi has been studied extensively since the middle of the previous century, however, our knowledge on the enzyme system used to degrade cellulose has changed dramatically just in the last three years. Traditionally, cellulose was thought to be degraded by three main types of enzyme activity: 1) endoglucanases (EC 3.2.1.4), 2) exoglucanases, including cellodextrinases (EC 3.2.1.74) and cellobiohydrolases (EC 3.2.1.91 for the non-reducing end acting cellobiohydrolases and EC 3.2.1.176 for the reducing end acting ones) and 3) β-glucosidases (EC 3.2.1.21) [11]. Endo-acting hydrolases introduce random breaks in the amorphous regions of the polysaccharide chain, exo-acting hydrolases cut processively cellooligosaccharides from chain ends and β-glucosidases hydrolyze soluble cellodextrins and cellobiose to glucose. In spite of the cooperative activity exhibited by the aforementioned biocatalysts, the impressive biomass degrading efficiency demonstrated by various microorganisms in nature cannot be solely attributed to this endo-exo hydrolytic mechanism. Extracting and processing a single cellulose chain from its compact environment is energetically demanding considering the high crystallinity of cellulose and its tight association to other cell wall polysaccharides. Systems releasing small molecular weight oxidants such as the hydroxyl free radical that randomly attack the substrate via Fenton type chemistry reactions have been thought to act in conjunction with common cellulases in lignocellulose degradation. These include cellobiose dehydrogenase, quinone redox cycling and glycopeptide-based Fenton reaction [12, 13].

Since the mid-20th century, researchers have suggested the presence of an additional non-hydrolytic factor that renders biomass less recalcitrant to enzymatic attack [14]. According to the proposed mechanism, cellulose hydrolysis was accomplished by the synergistic activity of two components, the first (C_1_) swelling and disrupting cellulose and the second (C_x_) having endoglucanase activity. In spite of many years of research, the nature of component C_1_ has long remained an unresolved issue [15]. Previous studies have suggested components such as carbohydrate binding modules, expansins and expansin-like proteins (e.g. swollenin) as potential candidates for the C_1_-mediated disruption of highly-ordered cellulose matrix [16]. A more recent study complemented this list with some fungal proteins with homology to glycosyl hydrolase (GH) family 61 of the continuously updated Carbohydrate Active enZyme database (CAZy; http://www.cazy.org), exhibiting cellulolytic enhancing ability when combined with common cellulases [17]. Interestingly, most of these proteins share a potential carbohydrate-binding surface; the exact mechanism, however, that renders recalcitrant lignocellulosic biomass accessible to degrading enzymes is yet to be fully elucidated.

## 2. GH61s: a cellulase-enhancing factor

To date, GH family 61 comprises approximately 250 members, widely distributed in the genome of most ascomycetous and basidiomycetous (white-rot and brown-rot) fungi [18, 19]. Expression levels of most GH61 genes increase considerably during growth on lignocellulosic substrates, as compared to glucose media, suggesting their active involvement in cellulose decomposition. [20]. Even though the existence of these proteins has been long known, it was not until very recently that their physiological function was unraveled. Initial studies on GH61s reported a weak endoglucanase activity that could not be considered as their main role *in vivo* [21, 22]. In 2007, it was reported that some GH61 members could boost cellulase activity resulting in increased lignocellulose conversion [17]. These findings launched intensive research efforts towards understanding the function of this enigmatic family. In 2010, *Harris et al*. identified three *Thielavia terrestris* GH61s as potential cellulase-enhancing factors [23]. The same group incorporated a *Thermoascus aurantiacus* GH61 encoding gene (*Ta*GH61A) in the genome of *Trichoderma reesei*, a common cellulase producer, resulting in a strain with improved cellulolytic efficiency. More precisely, the protein loading required to degrade lignocellulosic biomass was reduced two-fold [23]. It was also reported that this cellulase-boosting function was metal-ion dependent and eliminated when the mixture of cellulases/GH61 was applied on substrates composed solely of cellulose. One step further, the synergistic effect exhibited by *St*Cel61a, a GH61 from *Myceliopthora thermophila* (synonym *Sporotrichum thermophile*) was related to the lignin content and the antioxidant activity of an array of lignocellulosic materials [24]. Several hypotheses were put forward to explain GH61 mechanism such as the targeting of an unknown bond found in lignocellulose, but no definite answer was given regarding the interpretation of the enhancing effect.

**Figure 1:**
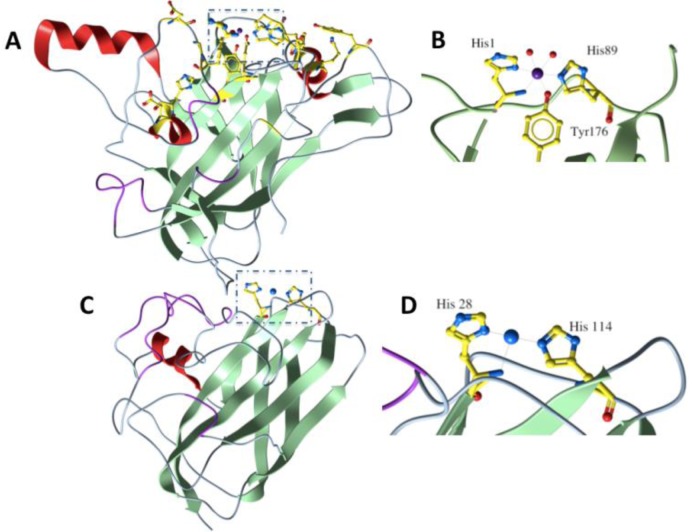
**A.** The figure shows the structure of Cel61B (molB, PDB code 2VTC) in cartoon representation. Conserved residues on the surface of the molecule are shown in ball and stick representation. **B**. The nickel ion (purple sphere) coordinated by His1, His 89, Tyr 176 and two water molecules (red spheres) in Cel61B structure. **C**. The structure of CBP21 (molC, PDB code 2BEM) in cartoon representation. Highlighted in ball and stick are the highly conserved residues His114 and His28, and a bound sodium ion (blue sphere). **D**. The sodium ion (blue sphere) coordinated by His28 and His 114 in molC of CBP21 structure. All figures were prepared with Molsoft [27].

The first crystal structure of a GH61 member, Cel61B from *Hypocrea jecorina* (anamorph *T. reesei*) was determined in 2008 at 1.6 Å resolution [25], followed by the 1.9 Å structure of *T. terrestris* GH61E [23]. Both GH61s fold into a beta-sandwich, where the two antiparallel twisted beta-sheets are connected through loops of varying length and conformation. The majority of conserved residues are clustered on the surface of the protein (Figure 1A). Cel61B structure comprises three nickel ions located in the two molecules of the asymmetric unit. Two of them are near the N-terminal of the two monomers and coordinated by highly conserved residues among GH61 family members (His1, His 89 and Tyr 176) (Figure 1B). In the case of GH61E, the corresponding ions are zinc or magnesium. In both structures, the authors did not manage to locate any polysaccharide binding cleft or typical glycoside hydrolase active site. A structural comparison search revealed that the most similar structure was that of CBP21 from *Serratia marcescens* [26], a protein that can be classified in carbohydrate-binding module family 33 (CBM33) of CAZy database and is known to be implicated in chitin degradation (Figure 1C). Interestingly, the two histidines coordinating the metal ion in GH61s superimposed nicely with the two highly conserved histidine residues in CBP21 structure (Figure 1D). It was suggested that GH61s and CBP21 could share a similar, even though at that time unknown, mechanism of action that led to increased hydrolysis rates of recalcitrant polysaccharides.

## 3. CBP21 – oxidative cleavage of chitin

Chitin is a crystalline analogue of cellulose composed of β-(1,4) linked units of *N*-acetyl-D-glucosamine (GlcNAc). It is widely distributed in nature, particularly in the cuticle of arthropods and the cell walls of fungi and yeast. Similarly to cellulose-degrading enzymes, chitinases can be divided into two major categories: endochitinases that cleave chitin randomly at internal sites and exochitinases that involve chitobiosidases and β-(1,4) *N*-acetyl glucosaminidases [28]. CBM33 proteins were originally thought to be involved in substrate recognition due to the fact that they were secreted upon growth on lignin, bound on it and had no detectable hydrolytic activity [29]. More recently, it was shown that CBM33s such as CBP21 from *S. marcescens* could boost the hydrolytic activity of chitinases, indicating a more active involvement in chitin degradation [30]. However, the exact enzyme mechanism remained elusive until 2010, when in a landmark study Vaaje-Kolstad et al. showed that CBP21 was actually an oxidase [31]. More precisely, the authors discovered that when applied on β-chitin, CBP21 released even-numbered oligosaccharides, oxidized at the reducing end, and that this activity increased dramatically upon addition of reductants such as ascorbic acid. CBP21 seemed to attack every second glycosidic bond, and taking into account that the repeating unit of the substrate is a disaccharide, it was suggested that this enzyme could act processively from one chain end towards a specific direction. Isotope labeling experiments showed that one of the oxygens inserted at the oxidation site came from water while the other one from molecular oxygen. Experimental evidence also demonstrated that divalent ions were necessary for CBP21 activity. In spite of this, the atomic structure at 0.95 Å resolution of a CBM33 from *Enterococcus faecalis*, *Ef*CBM33A, determined in a subsequent study, did not include any metal ion coordinated by the typical motif of conserved histidines at the N-terminal of the protein [32]. When applied on chitin with the addition of an external electron donor, such as reduced glutathione or ascorbic acid, *Ef*CBM33A released even-numbered chitooligosaccharide aldonic acids and enhanced substrate degradation in the presence of a GH18 chitinase. Finally, CelS2, another CBM33-containing protein from *Streptomyces coelicolor* A3, released even-numbered oxidized cellooligomers when applied on avicel and contributed to increased cellulose conversion when combined with common cellulases [33]. In summary, CBM33 proteins were shown to potentiate both cellulose hydrolysis by cellulases and chitin hydrolysis by chitinases, using an unprecedentent mechanism of oxidative chain cleavage.

## 4. GH61s: Polysaccharide Monooxygenases, PMOs

The structural similarity, common enhancing effect, as well as the fact that genes encoding GH61 proteins are uniquely found in fungi, while CBP21-like genes are widespread amongst bacteria and viruses [34], led to the assumption that GH61s are fungal counterparts of CBM33s, sharing the same oxidative mechanism. The landmark findings for CBP21 launched a series of studies showing, as anticipated, that GH61s function in a similar way on cellulose.

**Figure 2:**
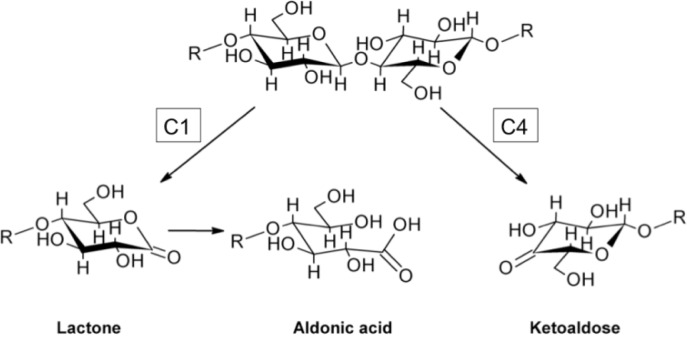
Oxidized reaction products released from GH61s applied on cellulosic substrates.

A GH61 from *T. aurantiacus*, *Ta*GH61, increased the conversion of microcrystalline cellulose by common cellulases only when gallic acid was present in the reaction mixture [35]. Analysis of *Ta*GH61 reaction products showed that this enzyme produced C1 oxidized cellooligomers, as well as non-reducing end oxidized species. Isothermal titration calorimetry, electron paramagnetic resonance, X-ray crystallography and reactivity experiments confirmed that GH61s bear a type II copper site, where the ion is coordinated by the nitrogen atoms of two highly conserved histidines. In addition, the 1.25 Å crystal structure of *Ta*GH61 revealed a methyl group covalently attached to atom Nɛ2 of the N-terminal histidine that had not been modeled in the two previous GH61 structures (*H. jecorina* Cel61B and *T. terrestris* GH61E). Even though this histidine is highly conserved and contributes to copper-ion coordination, the biological role of its methylation remains elusive. *Pc*GH61D, one of the 13 GH61 enzymes present in *Phanerochaete chrysosporium* genome [36], was also shown to oxidatively cleave cellulose [37]. Its activity was boosted upon addition of reductants such as ascorbic acid, glutathione and gallic acid. After treatment with EDTA, only copper and manganese were able to reactivate *Pc*GH61D. In the same report, it was also shown that the weak endoglucanase activity previously observed for some members of GH61 family could not be the outcome of a side-activity. The native oligosaccharides released in small amounts contained reducing ends that were already present in the original substrate, possibly resulting from oxidative cleavage close to the chain ends. Three *Neurospora crassa* GH61s also released a variety of oxidized species when applied on phosphoric acid swollen cellulose (PASC) [38]. Some GH61s were shown to oxidize glucose at position C1, releasing lactones that are either spontaneously or enzymatically hydrolyzed to aldonic acids [39], while others acted on the non-reducing end producing ketoaldoses that exist as different hydrated isomers in aqueous solution [40] (Figure 2).

**Figure 3:**
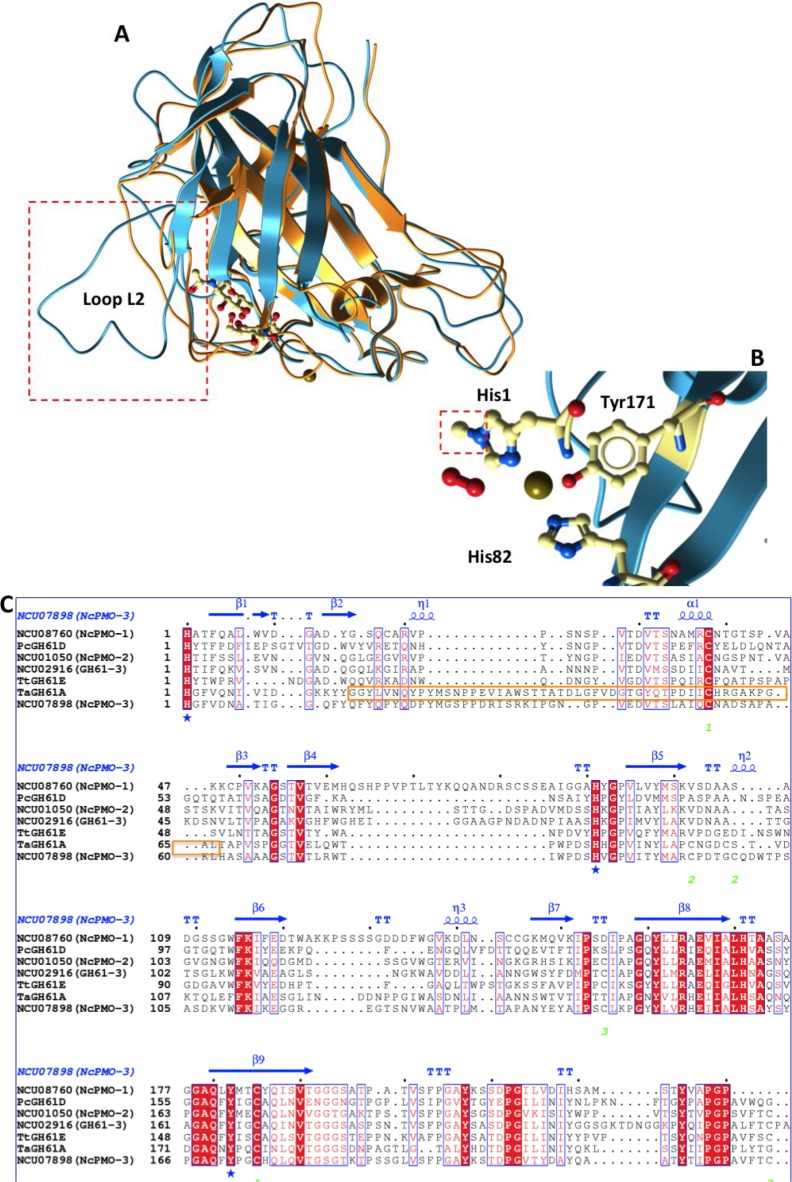
**A.** Superposition of the crystal structures of PMO-2 (PDB code 4EIR, molA) in orange and PMO-3 (PDB code 4EIS, molA) in cyan. Loop L2 is highlighted by a dashed square and the additional glycosylation site on PMO-3 is shown in ball and stick representation. **B**. Copper coordination site of PMO-3. The methylation site on the N-terminal histidine is shown in a dashed square. **C**. Multiple sequence alignment of PMO enzymes categorized in three types according to sequence, structural and biochemical characteristics (based on [37, 41]: Type 1: NCU08760 (*Nc*PMO-1), *Pc*GH61D, *Tt*GH61E, type 2: NCU01050 (*Nc*PMO-2), NCU02916(GH61-3) and type 3: NCU07898 (*Nc*PMO-3), *Ta*GH61A. Blue asterisks mark copper-coordinating residues. The secondary structure elements shown in blue and disulphide bonds (numbers in green) were assigned based on the crystal structure of *Nc*PMO-3 (PDB code 4EIS). Residues in the orange frame form loop L2. Identical and similar residues are printed in white on a red background and in red on a white background, respectively. Secondary structure elements α-helices, 3_10_-helices, β-strands and strict α and β-turns are denoted as α, η, β, TTT and TT, respectively. Multiple sequence alignment of homologous enzymes was performed with Clustal Omega [42] at EBI server and visualized with ESPript 2.2 [43]. Figures were prepared with Molsoft.

All the aforementioned findings indicated clearly that GH61s were erroneously classified as hydrolases; Phillips et al. suggested that they are re-categorized into a new class of carbohydrate-active enzymes called polysaccharide monooxygenases (PMOs) [38]. The same group subsequently divided PMOs into at least three types, based on their primary structure and substrate specificity [41]. Type 1 (PMO-1) hydroxylate the C1 position of the glucose moiety, type 2 (PMO-2) are specific for C4, while type 3 exhibit weaker specificity, releasing both reducing and non reducing-end oxidized products. The crystal structures of a type 2 and a type 3 PMO from *N. crassa* were determined in an effort to locate the structural determinants that affect substrate specificity [41]. Similarly to the previously determined structures, both enzymes fold into an immunoglobulin-like beta sandwich whose strands are connected by 8 loops with 2 or 3 alpha-helix insertions. The loops on the one side of the sandwich form a rather flat surface in the center of which is located the active site with the coordinated copper ion. Loop L2 is the most variable among PMOs, both in length and secondary structure (Figure 3A and 3C). PMO-3 feature both a glycosylation site and an extended conformation of loop L2 bearing a short 3-10 helix on the flat surface which are absent on type 2. These two structural features could be considered as substrate specificity determinants as they lie on top of the copper center. In both structures the N-terminal histidine is methylated, without though attributing a specific function to this methyl group (Figure 3B). In addition, one of the axial coordination sites of copper ion is occupied by a superoxide and a peroxide ion in the structure of PMO-2 and PMO-3, respectively.

Isotope-labeling experiments corroborated PMO specificity results [44]. ^18^O (from ^18^O_2_) was incorporated into the aldonic acid products of a PMO-1 using various reductant agents. The non-reducing end product of a PMO-2 (C4 or C6) was also identified by excluding C6 oxidation through the absence of glucuronic acid after oxidation with hypoiodite. The inclusion of oxygen at position C4 was confirmed by the formation of galactose after borohydride reduction of the hydrolyzed type-2 reaction products. According to the authors, cellulose cleavage at position 1 or 4 is energetically favored since it occurs through a simple elimination reaction, while oxygen insertion at other sites would require the cooperation of additional amino acids for glycosidic bond cleavage.

The aforementioned findings corroborated the oxidative mechanism of the enzymes previously known as GH61s. The terms PMO or LPMO, which stands for (Lytic) Polysaccharide MonoOxygenase, are currently widely accepted by the scientific community and used to refer to for this family of oxidases ([34, 45].

## 5. The redox-active cofactor – cellobiose dehydrogenase

**Figure 4:**
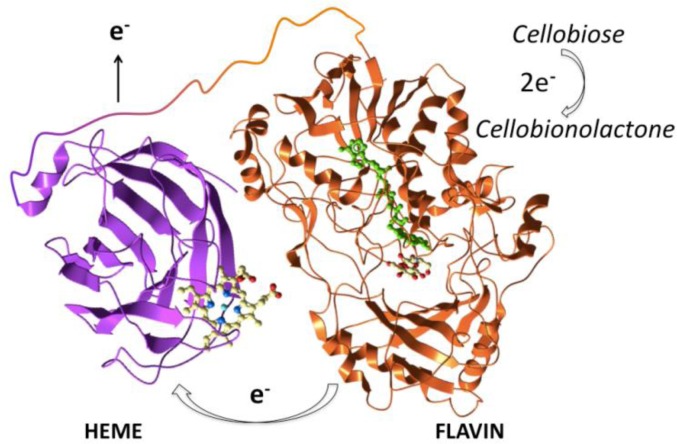
Schematic representation of a CDH comprising a C-terminal flavin domain with its FAD highighted in green (PDB code 1NAA, [51]) and an N-terminal heme domain (PDB code 1D7B, [52]. The protein structures were visualized with Molsoft [27]. Oligosaccharide oxidation takes place at the flavin domain followed by electron transfer to the ferric heme group.

In order to function efficiently, PMOs require a substance acting as electron donor. In the aforementioned studies, this factor is attributed to a reducing agent naturally occurring in the substrate (gallic acid, lignin), added externally (ascorbic acid, glutathione), but can also be a cosecreted enzyme, such as cellobiose dehydrogenase (CDH). CDH, the only known example of secreted flavocytochrome [46], is found in the genome of most wood-degrading fungi (white-rot, soft-rot and at least one brown-rot). All known CDH enzymes are composed of an N-terminal heme domain, which carries a cytochrome *b* type heme and a C-terminal flavin domain which contains FAD, connected by a flexible linker (Figure 4)[47]. Some of them also contain a C-terminal CBM, such as the CDH from *M. thermophila* [48]. They catalyze the reducing end oxidation of cellobiose, cellodextrins or other oligosaccharides to the corresponding lactones that are subsequently converted to their aldonic acids. The most effective electron donor is cellobiose, while the electron acceptor that they employ in nature, as well as their physiological function remain unknown [49]. The suggested roles for CDH are diverse, with the most widely proposed in literature being the contribution to lignocellulose degradation by generating hydroxyl radicals in a Fenton-type reaction [50]. However, the relevant catalytic efficiency is very low under *in vivo* conditions. The members of CDH family exhibit high sequence diversity and are divided based on phylogenetic criteria to three classes: I (from basidiomycetes), II and III (both from ascomycetes). Class II is further subdivided to IIA and IIB depending on the presence or not of a CBM, while the secretion of class III CDHs has not been verified experimentally. As far as substrate preference is concerned, the difference between class I and II CDHs is that class II CDHs also oxidize mono- and oligo-saccharides, with a reduced though specificity as compared to cellobiose [47].

Recent work has demonstrated that CDHs can act synergistically with PMOs in cellulose hydrolysis [53], by coupling cellobiose oxidation to the reductive activation of PMOs. Increased cellulose conversion was observed when a *T. aurantiacus* PMO (*Ta*GH61) and a *Humicola insolens* CDH (*Hi*CDH) were combined with common cellulases and applied on both crystalline and amorphous cellulosic substrates [54]. The binary combination of *Ta*GH61 and *Hi*CDH cleaved cellulose into a mixture of DP2-DP10 soluble oligosaccharides, comprising both reducing end-C1 oxidized species and non-reducing end modified oligosaccharides. When *cdh1* gene was deleted from *N. crassa* genome, the resulting strain exhibited reduced cellulolytic activity that was restored to wild type levels upon addition of purified CDH [38]. The enhancing effect was eliminated when EDTA was added and was also dependent on the presence of oxygen. In another study, two CDHs from *N. crassa*, CDH IIA and CDH IIB and one PMO, GH61-3 (NCU02916) were produced in *P. pastoris* [53]. CDH-PMO interaction was highlighted via the pH dependent inhibition of cytochrome *c* activity. Cyt c and PMO are competing substrates as receptors of the electrons released from CDH cytochrome domain. It was also shown that CDHs can accept electrons from different oligosaccharides such as xylooligosaccharides and interact with a variety of PMOs, suggesting that these enzymes may be implicated in the degradation of both cellulose and hemicellulose [55]. Finally, a *T. terrestris* CDH acted synergistically with a PMO but also with a β-glucosidase providing a role for these enigmatic enzymes in fungal lignocellulose degradation [56].

The concerted activity of PMOs and CDHs in oxidative cleavage of cellulose should not be overestimated, since not all organisms have genes encoding for both enzymes in their genomes [34, 57]. However, it is undoubted that CDHs are key biocatalysts that have to be considered when designing an artificial coctail for the efficient saccharification of plant biomass. A recent study demonstrated the role of two recombinant CDHs from *Coprinopsis cinerea* and *Podospora anserina* in the saccharification of wheat straw. According to the reported results, the addition of both CDHs to *T. reesei* secretome resulted in a decrease in reducing sugars released, but to varying degrees depending on the nature and the amount of the enzyme added [58]. The authors suggest that the observed reduction could be attributed to the oxidation of cleaved oligosaccharides to their lactones and that the production of H_2_O_2_ by CDHs could also have an effect on the lignocellulosic degradation.

## 6. Sequence diversity

In spite of the intensive research efforts launched after the discovery of the oxidative mechanism of PMOs, the issue of substrate specificity remains largely unanswered, but it is well established that the various members of this family display different substrate preferences. All substrates used in the relevant studies differ from natural cellulosic materials due to chemical or mechanical pretreatment, rendering even more difficult the identification of the natural bonds targeted by this group of enzymes. PMOs exhibit a striking diversity in their primary structure, largely maintained even before the divergence of the two major fungal phyla, *Ascomycota* and *Basidiomycota*, more than 600 million years ago [23]. The genome of *Heterobasidion irregulare*, a pathogenic white-rot fungus, involves 10 PMO coding genes. Interestingly, among the 10 *Heterobasidion irregulare* PMOs, one of them does not possess the conserved metal-binding motif. Multiple sequence alignment shows that they can be divided into groups based on the presence of an insert near the N-terminus and another near the second catalytic histidine, both potentially contributing to substrate binding interaction. The transcription profile of these PMOs also presents high variability, with one group of PMOs being up-regulated on woody substrates in tandem with other cellulases, while the other showing either down-regulation or unaltered gene expression compared to a control medium [59]. A comparative analysis of the genomes of two thermophilic fungi, *M. thermophila* and *T. terrestris* revealed that unlike the well-studied mesophile, *T. reesei*, both fungi harbor large numbers of PMOs, classified into 25 orthologous clades. It is suggested that that this clear expansion may be indicative of the evolution of alternative biomass degradation mechanisms by these fungi [60]. The high sequence diversity and differential regulation both point towards the need for different polysaccharide oxidases acting on different types of wood cell-wall compounds.

## 7. Summary and outlook

**Figure 5:**
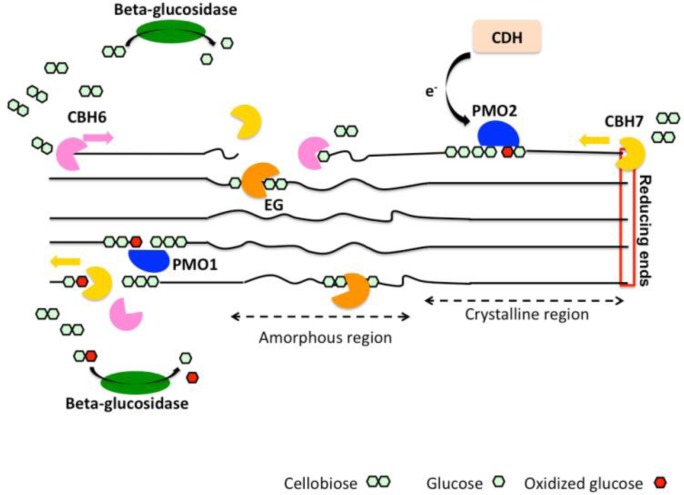
A simplified scheme of the current view on the enzymatic degradation of cellulose, involving cellobiohydrolases (CBH), endoglucanases (EG), type1 and type 2 PMOs (PMO1 and PMO2, respectively). Cellobiose dehydrogenase (CDH) is a potential electron donor for PMOs. EGs and PMOs cleave internally cellulose chains releasing chain ends that are targeted by CBHs. CBHs generate cellobiose or oxidized cellobiose that are subsequently hydrolyzed by β-glucosidase.

Today, it is widely accepted that a microbial oxidoreductive cellulose degrading system exists in parallel with the long-known hydrolytic cellulase system (Figure 5). There is no antagonistic relation between the two systems, since copper oxidases seem to attack the highly crystalline regions of cellulose in contrast to endoglucanases that are active on amorphous parts and cellobiohydrolases that require a cellodextrin chain end in order to initiate the processive crystalline cellulose cleavage. Even though the exact mechanism of action remains to be determined, it seems that PMOs and CBM33s carry out the action of C_1_ component suggested by Reese et al. by disrupting the tightly packed cellulose chains and rendering them more accessible for hydrolytic cellulases [61]. It is noteworthy that in spite of their structural homology, PMOs differ from CBP21 and CelS2 in that the products of these CBM33s are even-numbered C1-oxidized oligosaccharides, whereas the products released from PMOs present a higher diversity. Undoubtedly, the classification of PMOs and CBM33s as glycoside hydrolases and carbohydrate-binding modules, respectively, is no longer valid and should be reconsidered.

The discovery of these oxidative enzymes is of increased significance from a scientific and an industrial point of view. Unraveling a novel enzymatic mechanism will widen our understanding of cellulose digestion in nature and will contribute to the development of more efficient cocktails for low cost lignocellulosic biomass conversion. However, there are many scientific and technical issues that need to be addressed. In a recent study, a PMO containing commercial enzyme mixture produced by Novozymes, Cellic CTec2, was applied on pretreated wheat straw under conditions as close as possible to a bioethanol production setup. The cellulose conversion yield was significantly increased as a result of the presence of oxidative enzymes in the cellulolytic cocktail. However, it was shown that compared to glucose, gluconic acid had an increased inhibitory effect. Also, its production was affected negatively by temperature, providing guidelines for process design [62]. Undoubtedly, further work is needed for the effective introduction of oxidative enzymes in biotechnological applications. Current accumulated work on practical aspects of PMO use is rather limited. Research efforts should primarily focus on shedding light into the substrate specificity of different PMOs, potentially revealing their involvement in the degradation of other than cellulose cell wall components. The role of the methyl-modification observed on the N-terminal histidine should also be further investigated in terms of enzyme stability and catalytic function. For the application of oxidative biocatalysts in industrial bioethanol production, the amount and nature of the reducing agent added, as well as enzyme ratios (hydrolases versus oxidases), should be optimized for the maximal cellulose conversion rate while keeping enzyme loading as low as possible. The composition of the optimum blend should also be dictated by the composition of the specific feedstock and the characteristics of the process.

The discovery of the oxidoreductive cellulolytic system that acts complementarily to the better-described cellulolytic system has changed drastically our view of the enzymatic degradation of plant biomass. Efforts should be oriented towards reducing the enzyme loading and processing times by developing new strategies based on the properties of these novel biocatalysts. This will contribute to the production of cheap sugars and commercially competitive bioethanol, a long-pursued goal of biofuel industry.
